# Insights into the Predictors of Attitude toward Entomophagy: The Potential Role of Health Literacy: A Cross-Sectional Study Conducted in a Sample of Students of the University of Florence

**DOI:** 10.3390/ijerph18105306

**Published:** 2021-05-17

**Authors:** Chiara Lorini, Laura Ricotta, Virginia Vettori, Marco Del Riccio, Massimiliano Alberto Biamonte, Guglielmo Bonaccorsi

**Affiliations:** 1 Department of Health Sciences, University of Florence, v.le Morgagni 48, 50134 Florence, Italy; chiara.lorini@unifi.it (C.L.); laura.ricotta@aslroma1.it (L.R.); guglielmo.bonaccorsi@unifi.it (G.B.); 2 School of Specialization in Hygiene and Preventive Medicine, University of Florence, v.le Morgagni 48, 50134 Florence, Italy; marco.delriccio@unifi.it (M.D.R.); massimilianoalberto.biamonte@unifi.it (M.A.B.)

**Keywords:** nutrition, food literacy, food security, environment, survey, entomophagy

## Abstract

In Western countries, one of the main barriers to entomophagy is repulsion toward insects. Few studies have investigated the factors that influence attitudes toward entomophagy. Therefore, we conducted a cross-sectional study involving a sample of 248 university students, focusing on disgust and other potential attributes that can influence insect consumption, including health literacy. We used a 17-item self-administered questionnaire. Consistent with the literature, two items were chosen as outcome variables to evaluate the predictors of the propensity to consume insects: “Have you ever eaten insects or insect-based products?” and “How disgusting do you find eating insects?” The data analysis shows that having already eaten insects is inversely associated with the level of disgust (OR: 0.1, *p* < 0.01); and it is positively associated with higher levels of health literacy (OR: 3.66, *p* > 0.01). Additionally, having some knowledge and information about entomophagy is inversely associated with a higher level of disgust (OR: 0.44, *p* = 0.03 and OR: 0.25, *p* = 0.03, respectively), while being female is positively associated with disgust (OR: 3.26, *p* < 0.01). Our results suggest the potential role of health literacy, in addition to other factors, in influencing the willingness to taste insects. However, further studies involving larger and non-convenience samples are needed to confirm our hypothesis.

## 1. Introduction

The world population continues to grow exponentially (in 2050, the Earth will probably be home to 9 billion people), and current food resources, especially in terms of protein intake, will not be sufficient to sustain global requirements [1]. Consequently, it has become urgent to introduce, to daily nutrition, foods with nutritional values comparable to those of meat but requiring less energy to produce and having a smaller impact on the environment [2]. Insects stand out as possible alternatives, and in 2013, the Food and Agriculture Organization of the United Nations published a dedicated report named “Edible Insects” [3]. This report shows that insects can be a good source of fat, protein, vitamins, fibers, and minerals. On the other hand, the literature provides evidences related to potential risks in the consumption of edible insects [4], which are mainly related to microbial contamination, accumulation and production of chemical contaminants such as toxins and heavy metals, and allergenicity through inhalation or food ingestion by workers and consumers [5,6].

Although the European Food Safety Authority (EFSA) stated that the microbiological risks linked to the consumption of insects could be similar to the ones associated with other sources of unprocessed proteins [7], there are still many important issues to consider, such as to certify which processing technique neutralizes pathogenic organisms or how to avoid a cross-contamination [8].

Entomophagy traditions and culinary cultures are practiced worldwide and there is also an increasing interest in insects breeding [9]. However at present, introducing this custom in Europe remains challenging [10]. The main obstacle seems to be a repulsion toward a food that is considered to be disgusting and dangerous to human health [11]. The feeling of disgust is a complex phenomenon that, according to some authors, could be associated with the health risks posed by the consumption of a specific substance. On the other hand, it can be connected to advantages determined by the survival of animals, as in the case of cattle in India from which milk and dairy products are obtained [12]. Disgust toward a specific food is generally a collective dimension and is tightly linked to the culture of a population. Moreover, it is interesting to note that foods deemed disgusting or good to eat differ worldwide. Furthermore, disgust is not a static feeling, since what is convenient to eat can change over time, and people can change their food preferences through information exchange and experiences [12]. The possibility of commercializing insects in the European food market was introduced with European Regulation n. 2238/2015, which identifies them as “novel food”, meaning “any food that was not used for human consumption to a significant degree within the Union before 15 May 1997” [13,14]. It also includes foods with a “history of safe food used in a third country, meaning that the safety of the food in question has been confirmed with compositional data and from experience of continued use for at least 25 years in the customary diet of a significant number of people in at least one third country”. Northern European countries such as Belgium, Denmark, and the United Kingdom have started to open up toward this market.

Regarding the willingness to eat insects, some recent researches have investigated some characteristics of the consumers that are related to entomophagy [15], while other authors published an instrument capable of measuring the association between the characteristics of the consumers and the consumption of insects [16]. From different studies, an association to certain factors, either individual or contextual, seems to emerge: age, gender, country of origin, available information about entomophagy, attendance of courses in food sciences, the methods of production of insect-based foods, disgust feeling, and previous experiences [10,17]. Among the studies focused on public opinion regarding the possibility of eating insects, some have specifically targeted university students. It is relevant to note that there is a greater willingness to try alternative foods in young adults familiar with foreign cultures and with a high level of education [15,18].

In general, literacy and level of education have been globally recognized as important determinants of health [19], and they seem to represent positive influencing factors of consumers’ attitudes toward an insect-based meal [10]. Linked to these aspects, a greater level of health literacy (HL), a factor that, to date, has never been investigated or related to the consumption of insects, could have a role in increasing this tendency. The concept of HL was first introduced in the United States in the 1970s by Professor Scott K. Simonds (1974) [20], and it was defined in 1988 by the World Health Organization (WHO) to the Health Promotion Glossary as “the cognitive and social skills which determine the motivation and ability of individuals to gain access to, understand, and use information in ways which promote and maintain good health” [21]. Data from the European survey on HL show that almost 50% of adults in eight European countries (Austria, Bulgaria, Germany, Greece, Ireland, The Netherlands, Poland, and Spain) have inadequate levels of HL [22]. This leads to less healthy decisions, risky behaviors, worse health conditions, more hospital admissions, and higher costs for the health system. In the Italian context, some comforting data have shown a high mean HL score in the population, with inadequate levels of HL in a relatively small proportion of the population [23]. Several authors have discussed the relationship between the specific form of HL, food resilience behavior, and attitudes [24,25,26,27] adopted by individuals; nevertheless, how and if HL could influence entomophagy willingness is still unexplored.

Based on some evidence that suggests a relationship between consumers’ attitudes toward eating insects and education [10] and considering that HL could be regarded as an outcome of health education [28], our hypothesis is that a relationship exists between HL and entomophagy. This hypothesis is also supported by many studies related to the determinants of food-decision making process and food well-being. In particular, the latter considers the approach to food decision-making process from a holistic point of view. In this sense, food availability, food policy, food socialization, food literacy, and food marketing are described as drivers of food well-being, as well as outcomes of the decisions that people make in sustaining a positive relationship with food [28]. In this perspective, health and food literacy can be viewed as the ability to use available nutritional, technological and environmental information to drive food choices of expert consumers, favoring those that are positive for both individuals and societies [11,29]. Moreover, psychological motivation and ability to change could drive the introduction of insects into a diet, as well as a more general food well-being [11,30], helping to overcome disgust feeling on neophobia; motivation is also included into the definition of health literacy of Sørensen et al. [31]: “health literacy is linked to literacy and entails people’s knowledge, motivation and competences to access, understand, appraise, and apply health information in order to make judgments and take decisions in everyday life concerning healthcare, disease prevention and health promotion to maintain or improve quality of life during the life course”.

Therefore, the aim of this study is to investigate several potential predictors of the attitude toward eating insects in a group of students from the University of Florence, with a specific focus on the potential role of their health literacy levels.

In particular, the novelty of the present research is to assess the role of health literacy as a potential influent factor of entomophagy.

## 2. Materials and Methods

A cross-sectional study was conducted on a convenience sample of students of the University of Florence (Italy) who were attending a master’s degree program in food sciences (*n* = 108) or in engineering and computer science (*n* = 150). The choice was made to include young individuals with different degrees of knowledge in the field of food and nutrition, since the literature provides evidence that this kind of knowledge influences familiarity with entomophagy [6,10,32,33,34]. Participants were selected among university students, which was consistent with previous research [18,33]. A total of 258 students participated in the research. Students were recruited from October 2018 to April 2019 in their classrooms or in the immediate proximity during lesson breaks. Data were collected using an ad hoc, self-administered, paper-and-pencil, two-section questionnaire, which was developed by the research team after selecting items that were found in the literature to be relevant for insect consumption [35,36,37,38,39,40,41,42,43,44,45,46]. A multidisciplinary research team of three experts (a medical doctor and expert in public health, a researcher in public health, and a food safety and prevention officer) developed the questionnaire based on a literature review; moreover, a small sample of 27 university students was consulted to assess whether the questionnaire was easily understandable and sufficiently clear. Students’ comments helped to guide final adjustments of the first draft. The final version of the questionnaire in the Italian language is shown in the Supplementary file section.

The questionnaire included a general section investigating the socio-demographic characteristics of the sample: gender, age, country of birth [47,48,49], and eating habits [46,48]; this last question was used to exclude vegan or vegetarian students. Students involved in the survey were given some details about the research; however, consistent with Gere et al. [49], the fact that research dealt with entomophagy was omitted to avoid a high level of self-selection bias. The students were invited to voluntarily join the study. No informed consent was required, since data were collected anonymously. The study was conducted in accordance with the Helsinki Declaration. The questionnaires were digitized on site immediately after they were completed. Incomplete questionnaires and the questionnaires of those who declared their adoption of veganism or vegetarianism were excluded from the analysis, as were students from countries where entomophagy is a part of the culinary tradition, such as those in Africa, South America, Central and North America, Asia, and Oceania.

### 2.1. Questionnaire Design and Measures

The first section included four items with dichotomous answers (yes or no) related to knowledge of entomophagy [48], knowledge of the historical culinary tradition of entomophagy worldwide, and attitude toward the consumption of insects. Moreover, students were asked whether they had ever eaten insects or products that contained insects, and if so, what they had eaten [18,37,39], if they would recommend this experience to friends and relatives, if they liked them, and the reasons that could explain this preference [33,49]. In this section, there were also questions about the supposed advantages (nutritional composition, environmental impact, good taste, alternative to meat, and ease in finding insect-based products) [32,37,39,47,48,49,50] and the hypothetical disadvantages (cultural acceptance, allergic reactions, microbiological risks, and chemical risks). Participants were asked to assign a score to each advantage and disadvantage using a 5-points Likert scale according to the relevance that they attributed to each item (“many” = 4; “a lot” = 3; “sufficiently” = 2; “a few” = 1; “a little” = 0). The level of disgust derived from the consumption of insects was assessed with a specific question (“Zero to four, how disgusting do you find eating insects?”) using a 5-point Likert scale (“very disgusting” = 4; “disgusting = 3”; “slightly disgusting” = 2; “not very disgusting” = 1; “not disgusting at all” = 0).

Finally, the sample was asked to identify where they had heard about entomophagy [47], why they would consider the idea of starting to eat insects [33], the modalities (whole insects or flour) that they would prefer for consuming insects [37,39], and who, in their opinion, is responsible for the safety of insect-based products for human consumption. Those aspects were investigated using multiple-choice questions.

The second section consisted of six items to assess the students’ level of general HL. The level of HL was assessed through the short-short form of the European Health Literacy Survey Questionnaire (HLS-EU-Q6) [51]. Specifically, the previously validated Italian version of the HLS-EU-Q6 was used [52,53]. The responses to each item were given on a 4-point Agreement scale (“very easy” = 4; “fairly easy” = 3; “fairly difficult” = 2; “very difficult” = 1). Its final score was the mean value, which was taken into consideration only in cases in which the interviewees had answered at least five of the six items, and it varied between 1 and 4. The final score was used to classify HL into three levels: inadequate (score 1-2), problematic (2-3), and adequate (3-4) (the Italian version of the HLS-EU-Q6 is shown in the Supplementary file section).

### 2.2. Statistical Analyses

The collected data were entered into an ad hoc database. The percentage, median, and interquartile range (IQR) were calculated. In addition, for the two sections of questions related to the advantages and disadvantages of eating insects, the median was calculated for each subject as an overall summary trend index. The items that related to past experience (“Have you ever eaten insects or insect-based products?”) and that referred to disgust (“Zero to four, how disgusting do you find eating insects?”) were chosen, which was consistent with the scientific literature [10,33], as the most important predictors of the propensity to eat insects. The item related to disgust, previously expressed as a Likert scale score ranging from 0 to 4, was transformed into a binary variable, grouping answers 0, 1, 2, and 3 in order to stress the most “extreme” position (the maximum level of disgust). Normality was assessed using the Kolmogorov–Smirnov test. In order to assess the predictors of the two outcome variables (answers “yes” to the question “have you ever eaten insects or insect-based products?” and “4” to the item related to “disgust”), both univariate and multivariate analyses were performed. In particular, a univariate analysis was performed, including an evaluation of association using the Chi2 test or Fisher’s exact test for normally distributed variables and the Mann–Whitney U test for non-normally distributed continuous data. Variables that were found to be significantly associated were included in a backward stepwise procedure to perform two multiple linear regression analysis, one for each outcome variable. In particular, multiple logistic regression analysis was then performed with the item “Have you ever eaten insects or insect-based products?” as the outcome (dependent) variable, while the independent variables were the variables significantly associated in the univariate analysis, namely, “disgust”, the score on the HLS-EU-Q16, gender, age, and the medians of items related to advantages and disadvantages related to the consumption of insects. The approach was repeated using the “disgust” item as the outcome (dependent) variable, while the independent variables were gender, “heard about entomophagy”, “knowledge about historical culinary tradition of entomophagy”, the median of the items related to the advantages related to consumption of insects, and the median of the items related to the disadvantages related to consumption of insects. The Odds Ratio (OR) was used as a measure of association. For all of the analyses, a *p*-value of 0.05 was considered significant. The statistical analysis was performed using IBM SPSS Statistics for Windows, V.25.0 (IBM), and RStudio 1.2.5033.

## 3. Results

No student refused to complete the questionnaire. The final number of completed questionnaires was 248 after excluding 10 subjects because of incomplete answers or because they were vegans or vegetarians. No student was born in a country where entomophagy is a traditional habit. The sample was composed of students aged between 18 and 38 years old (median 23.0, IQR: 3.0); 132 (53.2%) were males, and the vast majority were Italians (96.8%). Only eight came from other countries (3.2%) (Albania, Kyrgyzstan, Romania, Morocco, Ukraine, and Gabon). Questionnaire answers and the univariate analysis between each item and the items chosen as predictors are shown in Table 1.

Most of the students (233, 94.0%) knew that eating insects is a common practice in many countries, although several had never heard about entomophagy (54, 21.4%). When considering the advantages that could come from the consumption of insects and insect-based products, the students judged the positive environmental impact as the most important one (median: 3.0; IQR: 2.0), which was followed by their nutritional composition (median: 2.0; IQR: 1.0). On the other hand, cultural acceptance (median: 3.0; IQR: 3.0) and microbiological hazards (median: 2.0, IQR: 1.0) were considered to be the most important disadvantages that could result from eating insects or insect-based products. For each subject, the median value of the responses to each of the two sections related to the advantages and disadvantages of eating insects, respectively, was calculated as an overall summary trend index. Both trend indexes showed a strong association with the answers related to the level of disgust: a higher median score of disadvantages was associated with a higher level of disgust (*p* < 0.01), and a higher median score of advantages showed an inverse relationship with the level of disgust (*p* < 0.01), while only the median of the disadvantages showed a positive association with the item “having eaten insects” (*p* < 0.05). Most of the participants had never tried insects or insect-based products (230, 92.7%). Among the subjects who had tried insects, some had eaten insect-based products such as biscuits, pancakes with cricket flour, ants, bugs, bagels, grasshoppers (both as a single food or inside a liqueur or with dark chocolate), scorpions, fried insects, and fried cockroaches. Moreover, when asked to specify the reasons why they might eat insects, most of them answered “for necessity” (96, 38.7%), while a similar proportion stated that they would do it to “try different flavors” (89; 35.9%). The results obtained from the question “how disgusting do you find eating insects?” highlighted that a group of students (79, 31.9%) was highly disgusted (score = 4) by the idea of eating insect-based products. Females presented a higher level of disgust (*p* < 0.01) in accordance with observations made by other authors [10]. One-third of the subjects was “inclined to follow suggestions take the advice of others trying insect-based products” (81, 32.7%), and this item was positively associated with both “having eaten insects” and a lower level of disgust (*p* < 0.01). The main reasons were related to tasting new flavors and foods or alternative foods.

According to the HLS-EU-Q6, 49 students had inadequate HL (19.8%), 147 students had problematic HL (59.2%), and 32 students presented sufficient HL (12.9%), while it was impossible to calculate the HL of 20 students (8.1%) due to missing responses. Students who had tried insects or insect-based products showed statistically significant higher levels of HL (*p* < 0.01). Two multiple logistic regression models were fitted using the item “Have you ever eaten insects or insect-based products?” (model 1) and the dichotomized item “disgust” (model 2) as dependent variables, respectively. Odds Ratios (ORs) are shown in Table 2. In model 1, a high level of disgust (answer “strong dislike”) showed a strong inverse association with the answer “yes, I have eaten insects or insect-based products” (OR 0.1, 95% CI 0.01–0.50, *p* < 0.01). In contrast, a high level of HL showed a positive association with the answer “yes, I have eaten insects or insect-based products” (OR 3.66, 95% CI 1.24–11.44, *p* > 0.01). In model 2, “having heard about entomophagy” and “having a previous knowledge about historical culinary tradition of entomophagy” had a strong inverse association with a higher level of “disgust” (OR 0.44, 95% CI 0.21–0.92, *p* = 0.03 and OR 0.25, 95% CI 0.07–0.92, *p* = 0.03, respectively); similarly, a higher median total score for the “advantages” items was inversely associated with a higher level of “disgust” (OR 0.57. 95% CI 0.41–0.78, *p* > 0.01). On the other hand, being female and having a higher median total score for the “disadvantages” items were positively associated with a higher level of disgust (OR 3.26, 95% CI 1.75–6.27, *p* < 0.01 and OR 1.45, 95% CI 1.02–2.10, *p* = 0.04, respectively) (Table 2).

In model 1, a high level of disgust (answer “strong dislike”) showed a strong inverse association with the answer “yes, I have eaten insects or insect-based products” (OR 0.1, 95% CI 0.01–0.50, *p* < 0.01). In contrast, a high level of HL showed a positive association with the answer “yes, I have eaten insects or insect-based products” (OR 3.66, 95% CI 1.24–11.44, *p* > 0.01). In model 2, “having heard about entomophagy” and “having a previous knowledge about historical culinary tradition of entomophagy” had a strong inverse association with a higher level of “disgust” (OR 0.44, 95% CI 0.21–0.92, *p* = 0.03 and OR 0.25, 95% CI 0.07–0.92, *p* = 0.03, respectively); similarly, a higher median total score for the “advantages” items was inversely associated with a higher level of “disgust” (OR 0.57. 95% CI 0.41–0.78, *p* > 0.01). On the other hand, being female and having a higher median total score for the “disadvantages” items was positively associated with a higher level of disgust (OR 3.26, 95% CI 1.75–6.27, *p* < 0.01 and OR 1.45, 95% CI 1.02–2.10, *p* = 0.04, respectively).

## 4. Discussion

Insects and insect-based products represent a potential optimal human food source, since they have positive nutritional and physiological characteristics. Several authors have explored the advantages and disadvantages that are connected to insect consumption [4,5,32,33,37,39,47,49,50], and disgust toward insects seems to be among the main barriers of their consumption in Western countries [18,32,35,39]. Disgust is conceptualized as an adaptive reaction: it is characterized by a collective dimension, and it is important for the protection of human health; nevertheless, it can be modified by information and life experiences [12,54].

In our study, we investigated several aspects related to eating insects and disgust toward this kind of food through a cross-sectional study involving a sample of 248 students of the University of Florence.

Additionally, we attempted to fill the gap in the literature that explores the role played by HL as a determinant of consumers’ attitudes toward insect-based products. Recently, in the Italian context, some studies have focused on the willingness to consume insects and the influencing factors of entomophagy [10,15,18,32,33]; furthermore, to previous research efforts, our results may be used to advance innovations that increase the willingness to attempt.

The university students included in our sample (*N* = 248) seem to have received some information about insect consumption. In fact, 94% had information about traditional food practices of eating insects in many countries, and 78.2% of the sample confirmed that they had heard about entomophagy.

Students involved in the study identified some of the main positive (advantages) as well as negative (disadvantages) factors associated with the consumption of insects. Advantages identified as most relevant were nutritional composition, environmental impact, and ease of identification; the main disadvantages were allergic reactions, microbiological risks, chemical risk, and cultural acceptance. Regarding real experiences in trying new kinds of food, most subjects in our sample had never tried insects or insect-based products (92.7% of the total sample). Additionally, a large part of the group (31.9%) declared being very disgusted by the idea of eating insects: among the 248 students, those who could image starting to eat insects would do it mainly if it was necessary (38.7%); additionally, 21.8% referred they would not eat insects at all.

The role of knowledge in influencing the willingness to attempt entomophagy is discussed in the literature, and Mancini et al. [10] recently highlighted the role of educational seminars and information provision about entomophagy. Specific information seems to contribute to empowering consumers’ attitudes toward eating insects and lowering food rejection [10]; the same authors also showed other influencing factors of intention in their framework. Despite the knowledge possessed by the majority of young students of the sample and their relative familiarity with entomophagy, many of them were disgusted by insects as food.

Some authors have regarded disgust in relation to entomophagy as an individual difference (i.e., disgust sensitivity or food disgust sensitivity) and as a descriptor of specific food (disgusting vs. tasty) [32,37]. These results are consistent with this study, as the students in Florence who described insects as disgusting were less likely to have reported eating them.

In the exhaustive framework conceptualized by Mancini et al. [10], the role of literacy in the field of health remains unexplored. In our perspective, in addition to other factors, HL could influence the willingness to eat insects or insect-based products. Individuals’ HL is reached when they become able to understand and use health information, and this contributes to the community’s proficiency in managing and defending their own and other people’s health [55,56]. Furthermore, in the field of food health, the evidence suggests that the adoption of the correct behavior and attitude is related to high levels of an individual’s literacy in this field [24,25,27]. Overall, the majority of our sample, despite being university students, showed problematic or inadequate levels of HL (59.2% showed a problematic level of HL, while 19.8% had an inadequate level of HL), and only 12.9% of the group had adequate HL. Interestingly, these results differ from some previous data obtained involving an adult population-based sample, which showed that 11.5%, 24.6%, and 63.9% of the sample had a high likelihood of limited HL, a possibility of limited HL, and adequate HL, respectively [23]. An inadequate level of HL suggests that individuals did not develop critical components that are useful for proficiently selecting and analyzing information and its quality. It is also possible to hypothesize that the environment in which our participants lived did not provide enough information about entomophagy, or access to insect-based products.

Disgust is closely connected to information possessed by individuals, as the literature has suggested [10]. Our results partially confirm the literature’s conceptualization, and, in particular, our analysis shows the significant role of disgust in predicting the propensity to eat insects or insect-based products. Mancini et al. [10] focused on the strong relationship between information and disgust, highlighting the role of educational seminars and information provision about entomophagy in empowering consumers’ attitudes toward eating insects and lowering food rejection. Additionally, the results obtained by the authors show that providing specific information (technological, social, and cultural contexts) could change the magnitude of disgust. The conceptualization provided by the literature is focused on disgust as one of the main factors influencing the willingness to eat insects.

## 5. Limits and Conclusions

Further studies are necessary to confirm the role of HL in influencing the propensity to eat insects or insect-based product, as suggested by our results. In particular, primary studies involving larger samples of individuals are needed. Therefore, along with the strengths highlighted above, one of the weaknesses of the present study could derive from the survey sample, which was limited and mainly based on convenience, because, at the time, the university student population was more easily available for consultation. Extending the research to a larger sample that is, representative of the general population and involves more cities or regions, thereby producing more reliable results, would more precisely determine the role of HL in the acceptance of new forms of nutrition, even at the expense of initial disgust.

The present paper represents the first attempt to investigate the described research question. In the future, the hypothesis presented here could be verified by stronger evidence. If our results are confirmed through a primary study involving a general population sample, it may be reasonable to plan educational programs that address students and focus on competences, with the aim of critically providing information regarding health and entomophagy. These kinds of educational programs have been proven to be effective in improving the capacity to find, analyze, interpret, and select information based on the scientific evidence [53]. Additionally, the capacity to process information could determine awareness regarding the possibility of inserting insects into a daily diet and considering them to be a safe and healthy food source of energy. In the end, it could favor the affirmation of entomophagy as a common practice for individuals involved in the intervention.

## Figures and Tables

**Table 1:** Questionnaire answer frequencies and univariate analysis between each item and the chosen predictors.

Sample Characteristics	Answers or Sub-Items	*N* (%) or Median (IQR)	Have Eaten Insects	*p* *	Showed Maximum Disgust (“Strong Dislike”)	*p* *
Age		23.0 (3.00)	23.0 (2.0)	>0.05	23.0 (2.0)	>0.05
Sex	M	132 (53.2%)	8 (6.1%)	>0.05	31 (23.5%)	**<0.01**
	F	116 (46.8%)	10 (8.6%)		48 (41.4%)	
**Questionnaire Items**	**Answers or sub-items**	***N* (%) or median (IQR)**	**Have eaten insects**	*p* *	**Showed maximum disgust**	*p* **
A. “Have you ever heard of entomophagy?”	Yes	194 (78.2%)	16 (8.3%)	>0.05	53 (27.3%)	**<0.01**
	No	54 (21.8%)	2 (3.7%)		26 (48.1%)	
B. “Where have you heard of entomophagy?”	Gastronomic events	22 (8.9%)	5 (22.7%)	**<0.01**	7 (31.8%)	>0.05
	University	57 (23.0%)	9 (15.8%)	**<0.01**	15 (26.4%)	>0.05
	Mass Media	148 (59.7%)	7 (4.7%)	**<0.01**	39 (26.3%)	**<0.01**
	Other	19 (7.7%)	4 (21.1%)	>0.05	5 (26.3%)	>0.05
C. “Do you know that there is an historical culinary tradition of entomophagy worldwide?”	Yes	233 (94.0%)	18 (7.7%)	>0.05	69 (29.6%)	**<0.01**
	No	15 (6.0%)	0		10 (66.7%)	
D. “What advantages could come from the consumption of insects?” (Likert scale 0-4 for each sub-item)	Nutritional compositions	2.0 (1.0)	3 (1.0)	>0.05	2.0 (1.0)	**<0.01**
	Environmental impact	3.0 (2.0)	3 (0.0)	>0.05	2.0 (2.0)	**<0.01**
	Alternative to meat	2.0 (2.0)	2.5 (2.8)	>0.05	1.0 (1.0)	**<0.01**
	Flavor	1.0 (2.0)	1.0 (1.7)	>0.05	0 (1.0)	**<0.01**
	Easy to find	3.0 (2.0)	3.0 (1.7)	>0.05	3.0 (1.0)	**<0.05**
	Median of the advantages	2.0 (1.0)	3.0 (1.0)	>0.05	2.0 (1.0)	**<0.01**
E. What disadvantages could come from the consumption of insects?” 0-4 for each sub-item)	Cultural acceptance	3.0 (3.0)	2.5 (2.0)	>0.05	3.0 (3.0)	>0.05
	Allergic reactions	2.0 (2.0)	2.0 (1.0)	**<0.05**	2.0 (1.0)	**<0.01**
	Microbiological hazards	2.0 (1.0)	2.0 (1.5)	>0.05	3.0 (1.0)	**<0.01**
	Chemical hazards	2.0 (1.0–3.0)	2.0 (1.0)	>0.05	2.0 (2.0)	**<0.01**
	Median of the disadvantages	2.0 (1.5–3.0)	2.0 (0.5)	**<0.05**	2.5 (1.0)	**<0.01**
F. “Have you ever eaten insects or insect-based products”?	Yes	18 (7.3%)	-	-	1 (5.6%)	**<0.01**
	No	230 (92.7%)	-		78 (33.9%)	
G. “Why might you start eating on insects?”	Necessity	96 (38.7%)	7 (7.3%)	**<0.01**	32 (33.3%)	**<0.01**
	Local tradition/habits	23 (9.3%)	3 (13.0%)	**<0.01**	2 (8.7%)	**<0.01**
	Try different flavors	89 (35.9%)	12 (13.5%)	**<0.01**	5 (5.6%)	**<0.01**
	Nutritional characteristics	50 (20.2%)	6 (12.0%)	**<0.01**	3 (6.0%)	**<0.01**
	Other reasons	7 (2.8%)	1 (14.3%)	>0.05	0	**<0.01**
	I would never try	54 (21.8%)	0	**<0.01**	42 (77.8%)	**<0.01**
H. “Zero to four, how disgusting do you find eating insects?”	Low (0-3)	169 (68.1%)	17 (10.1%)	**<0.01**	-	-
	High (4)	79 (31.9%)	1 (1.3%)		-	
I. “How do you prefer to eat insects?”	Whole insects	25 (10.1%)	2 (8.0%)	>0.05	2 (8.0%)	**<0.01**
	Powdered insects	110 (44.4%)	10 (9.1%)		25 (22.7%)	
	Both ways	41 (16.5%)	4 (9.8%)		0	
	In no way	72 (29.0%)	2 (2.8%)		52 (72.2%)	
L. “Would you recommend others to try insects or insect-based products?”	Yes	81 (32.7%)	13 (16.0%)	**<0.01**	5 (6,2%)	**<0.01**
	No	167 (67.3%)	5 (3.0%)		74 (44.3%)	
M. “In your opinion, which supervisory body should be responsible for controlling the production of insects for human consumption?”	Same authority assigned as supervisor for human consumption foodstuffs	169 (68.1%)	9 (5.3%)	>0.05	55 (32.2%)	>0.05
	Producers/producer association	9 (3.6%)	0		2 (22.2%)	
	International officers	19 (7.7%)	2 (10.5%)		4 (21.1%)	
	I don’t know	51 (20.6%)	7 (13.7%)		18 (35.3%)	
N. HLS-EU-Q6 **	Inadequate	49 (19.8%)	0	**<0.05**	16 (35.7%)	>0.05
	Problematic	147 (59.2%)	14 (9.5%)		46 (31.3%)	
	Sufficient	32 (12.9%)	4 (12.5%)		11 (34.4%)	
	NA	20 (8.1%)	0		6 (30.0%)	

* *p*-values were calculated using the Chi2 test or Fisher’s exact test when *n* < 5-for categorical variables and the Mann–Whitney U test for non-normally distributed continuous data (α = 0.05 and CI 95%). ** HL levels according to the HLS-EU-Q6 which is composed by following items: On a scale from very easy to very difficult, how easy would you say it is to (very easy, fairly easy, fairly difficult, very difficult, don’t know): 1—understand information in the media on how to get healthier?; 2—judge if the information on health risks in the media is reliable?; 3—judge which everyday behavior is related to your health?; 4—understand advice on health from family members or friends?; 5—use information the doctor gives you to make decisions about your illness?; 6—judge when you may need to get a second opinion from another doctor? The bold suggest that between those variables there is a significant association.

**Table 2:** Multivariate logistic regression models. Dependent variables: “Have you ever eaten insects or insect-based products?” answer (model 1) and “Zero to four, how disgusting do you find eating insects?” answer (model 2). With the latter, grouped answers (0–3 vs. 4) have been chosen to fit the model. The bold suggest that between those variables there is a significant association.

Variables	Estimate	Std. Error	OR	95% Confidence Intervals	*p*
**Model 1. Dependent variable: “Have you ever eaten insects or insect-based products?” (“yes” vs. “no”)**					
Age	−0.05	0.11	0.95	0.75–1.12	0.67
Sex-F	0.57	0.51	1.77	0.64–5.03	0.27
Disgust–“strong dislike”	−2.34	1.05	0.10	0.01–0.50	**<0.01**
Health Literacy–HLS-EU-Q6 (total)	1.30	0.56	3.66	1.24–11.44	**<0.01**
**Model 2. Dependent variable: “How disgusting do you find eating insects?” (“strong dislike” vs. “slightly dislike or not dislike at all”)**					
Sex-F	1.18	0.32	3.26	1.75–6.27	**<0.01**
Heard about entomophagy	−0.82	0.37	0.44	0.21–0.92	**0.03**
Knowledge about historical culinary tradition of entomophagy	−1.38	0.62	0.25	0.07–0.83	**0.03**
Advantages related to consumption of insects (median)	−0.56	0.16	0.57	0.41–0.78	**<0.01**
Disadvantages related to consumption of insects (median)	0.37	0.18	1.45	1.02–2.10	**0.04**

## Data Availability

The data are stored in a password-protected electronic archive held by the person in charge of the study. Only the person in charge of the study and the researchers of the present paper can access the file archive.

## Supplementary Materials

The following are available online at https://www.mdpi.com/article/10.3390/ijerph18105306/s1. Questionario sulle abitudini alimentary.

## References

[1] Hunter M.C., Smith R.G., Schipanski M.E., Atwood L.W., Mortensen D.A. (2017). Agriculture in 2050: Recalibrating targets for sustainable intensification. Bioscience.

[2] Klunder H.C., Wolkers-Rooijackers J., Korpela J.M., Nout M.J.R. (2012). Microbiological aspects of processing and storage of edible insects. Food Control..

[3] Van Huis A., van Itterbeeck J., Klunder H., Mertens E., Halloran A., Muir G., Vantomme P. (2013). Edible Insects. Future Prospects for Food and Feed Security.

[4] Cappelli A., Oliva N., Bonaccorsi G., Lorini C., Cini E. (2020). Assessment of the rheological properties and bread characteristics obtained by innovative protein sources (Cicer arietinum, Acheta domesticus, Tenebrio molitor): Novel food or potential improvers for wheat flour?. LWT.

[5] Cappelli A., Cini E., Lorini C., Oliva N., Bonaccorsi G. (2020). Insects as food: A review on risks assessments of Tenebrionidae and Gryllidae in relation to a first machines and plants development. Food Control..

[6] Sogari G., Mora C., Menozzi D. (2019). Edible Insects in the Food Sector—Methods, Current Applications and Perspectives.

[7] EFSA (European Food Safety Authority) (2015). Risk profile related to production and consumption of insects as food and feed. EFSA J..

[8] Raheem D., Raposo A., Oluwole O.B., Nieuwland M., Saraiva A., Carrascosa C. (2019). Entomophagy: Nutritional, ecological, safety and legislation aspects. Food Res. Int..

[9] Raheem D., Carrascosa C., Oluwole O.B., Nieuwland M., Saraiva A., Millán R., Raposo A. (2019). Traditional consumption of and rearing edible insects in Africa, Asia and Europe. Crit. Rev. Food Sci. Nutr..

[10] Mancini S., Moruzzo R., Riccioli F., Paci G. (2019). European consumers’ readiness to adopt insects as food. A review. Food Res. Int..

[11] Rozin P., Fallon A.E. (1987). A perspective on disgust. Psychol. Rev..

[12] Testa M., Stillo M., Maffei G., Andriolo V., Gardois P., Zotti C.M. (2016). Ugly but tasty: A systematic review of possible human and animal health risks related to entomophagy. Crit. Rev. Food Sci. Nutr..

[13] (1997). Regulation (EC) No 258/97 of the European Parliament and of the Council of 27 January 1997 concerning novel foods and novel food ingredients. Off. J. Eur. Union.

[14] (2015). Regulation (EU) 2015/2283 of the European Parliament and of the Council of 25 November 2015 on novel foods, amending Regulation (EU) No 1169/2011 of the European Parliament and of the Council and repealing Regulation (EC) No 258/97 of the European Parliament and of the Council and Commission Regulation (EC) No 1852/2001. Off. J. Eur. Union.

[15] Roma R., Ottomano Palmisano G., De Boni A. (2020). Insects as novel food: A consumer attitude analysis through the dominance-based rough set approach. Foods.

[16] La Barbera F., Verneau F., Videbæk P.N., Amato M., Grunert K.G. (2020). A self-report measure of attitudes toward the eating of insects: Construction and validation of the Entomophagy Attitude Questionnaire. Food Qual. Prefer..

[17] Toti E., Massaro L., Kais A., Aiello P., Palmery M., Peluso I. (2020). Entomophagy: A narrative review on nutritional value, safety, cultural acceptance and a focus on the role of food neophobia in Italy. Eur. J. Investig. Health Psychol. Educ..

[18] Cicatiello C., De Rosa B., Franco S., Lacetera N. (2016). Consumer approach to insects as food: Barriers and potential for consumption in Italy. Br. Food J..

[19] Whitehead M., Dahlgren G. (1991). What can be done about inequalities in health?. Lancet.

[20] Simonds S.K. (1974). Health education as social policy. Health Educ. Monogr..

[21] Nutbeam D., Kickbusch I. (1998). Health promotion glossary. Health Promot. Int..

[22] Sørensen K., van den Broucke S., Pelikan J.M., Fullam J., Doyle G., Slonska Z., Kondilis B., Stoffels V., Osborne R.H., Brand H. (2013). HLS-EU Consortium. Measuring health literacy in populations: Illuminating the design and development process of the European Health Literacy Survey Questionnaire (HLS-EU-Q). BMC Public Health.

[23] Bonaccorsi G., Lastrucci V., Vettori V., Lorini C. (2019). Functional health literacy in a population-based sample in Florence: A cross-sectional study using the Newest Vital Sign. BMJ Open.

[24] Carbone E.T., Zoellner J.M. (2012). Nutrition and health literacy: A systematic review to inform nutrition research and practice. J. Acad. Nutr. Diet..

[25] Doustmohammadian A., Omidvar N., Keshavarz Mohammadi N., Eini-Zinab H., Amini M., Abdollahi M., Amirhamidi Z., Haidari H. (2020). Low food and nutrition literacy (FNLIT): A barrier to dietary diversity and nutrient adequacy in school age children. BMC Res. Notes.

[26] Ruiz L.D., Zuelch M.L., Dimitratos S.M., Scherr R.E. (2019). Adolescent obesity: Diet quality, psychosocial health, and cardiometabolic risk factors. Nutrients.

[27] Vettori V., Lorini C., Milani C., Bonaccorsi G. (2019). Towards the implementation of a conceptual framework of food and nutrition literacy: Providing healthy eating for the population. Int. J. Environ. Res. Public Health.

[28] Scott M.L., Vallen B. (2019). Expanding the lens of food well-being: An examination of contemporary marketing, policy, and practice with an eye on the future. J. Public Policy Mark..

[29] Block L.G., Grier S., Childers T., Davis B., Ebert J., Kumanyika S., Russell N.L., Machin J.E., Motley C.M., Peracchio L. (2011). From nutrients to nurturance: A conceptual introduction to food well-being. J. Public Policy Mark..

[30] Bublitz M.G., Rajagopal P., Peracchio L.A., Peter P.C., Motley C.M., Scott M.L., Vallen B., Kees J., Andreasen A.R., Gelfand Miller E. (2013). Promoting positive change: Advancing the food well-being paradigm. J. Bus. Res..

[31] Sørensen K., van den Broucke S., Fullam J., Doyle G., Pelikan J., Slonska Z., Brand H. (2012). Health literacy and public health: A systematic review and integration of definitions and models. BMC Public Health.

[32] Sogari G. (2015). Entomophagy and Italian consumers: An exploratory analysis. Prog. Nutr..

[33] Sogari G., Menozzi D., Mora C. (2017). Exploring young foodies’ knowledge and attitude regarding entomophagy: A qualitative study in Italy. Int. J. Gastron. Food Sci..

[34] Sogari G., Amato M., Biasato I., Chiesa S., Gasco L. (2019). The potential role of insects as feed: A multi-perspective review. Animals.

[35] Caparros Megido R., Geuens M., Brostaux Y., Alabi T., Blecker C., Drugmand D., Éric Haubruge E., Frédéric Francis F. (2014). Edible insects acceptance by Belgian consumers: Promising attitude for entomophagy development. J. Sens. Stud..

[36] Hartmann C., Shi J., Giusto A., Siegrist M. (2015). The psychology of eating insects: A cross-cultural comparison between Germany and China. Food Qual. Prefer..

[37] Hartmann C., Siegrist M. (2016). Becoming an insectivore: Results of an experiment. Food Qual. Prefer..

[38] Hartmann C., Siegrist M. (2017). Insects as food: Perception and acceptance. Findings from current research. Ernahr. Umsch..

[39] Hartmann C., Siegrist M. (2018). Development and validation of the Food Disgust Scale. Food Qual. Prefer..

[40] Meyer-Rochow J., Paoletti M. (2005). Traditional food insects and spiders in several ethnic groups of Northeast India, Papua New Guinea, Australia and New Zeland. Ecological Implications of Minilvestock.

[41] Shelomi M. (2015). Why we still don’t eat insects: Assessing entomophagy promotion through a diffusion of innovations framework. Trends Food Sci. Technol..

[42] Schösler H., De Boer J., Boersema J.J. (2012). Can we cut out the meat of the dish? Constructing consumer- oriented pathways towards meat substitution. Appetite.

[43] Van Huis A. (2017). Edible insects and research needs. J. Insects Food Feed..

[44] Vanhonacker F., van Loo E., Gellynck X., Verbeke W. (2016). Flemish consumer attitudes towards more sustainable food choices. Appetite.

[45] Verbeke W. (2015). Profiling consumers who are ready to adopt insects as a meat substitute in a western society. Food Qual. Prefer..

[46] Yen A.L. (2009). Edible insects: Traditional knowledge or western phobia?. Entomol. Res..

[47] Balzan S., Fasolato L., Maniero S., Novelli E. (2016). Edible insects and young adults in a north-east Italian city an exploratory study. Br. Food J..

[48] Sidali K.L., Pizzo S., Garrido-Pérez E.I., Schamel G. (2019). Between food delicacies and food taboos: A structural equation model to assess Western students’ acceptance of Amazonian insect food. Food Res. Int..

[49] Gere A., Székely G., Kovács S., Kókai Z., Sipos L. (2017). Readiness to adopt insects in Hungary: A case study. Food Qual. Prefer..

[50] Pambo K.O., Okello J.J., Mbeche R.M., Kinyuru J.N., Alemu M.H. (2018). The role of product information on consumer sensory evaluation, expectations, experiences and emotions of cricket-flour-containing buns. Food Res. Int..

[51] Sørensen K., Pelikan J.M., Röthlin F., Ganahl K., Slonska Z., Doyle G., Fullam J., Kondilis B., Agrafiotis D., Uiters E. (2015). HLS- EU Consortium. Health literacy in Europe: Comparative results of the European Health Literacy Survey (HLS-EU). Eur. J. Public Health.

[52] Lorini C., Santomauro F., Grazzini M., Mantwill S., Vettori V., Lastrucci V., Bechini A., Boccalini S., Bussotti A., Bonaccorsi G. (2017). Health literacy in Italy: A cross-sectional study protocol to assess the health literacy level in a population-based sample, and to validate health literacy measures in the Italian Language. BMJ Open.

[53] Lorini C., Lastrucci V., Mantwill S., Vettori V., Bonaccorsi G. (2019). Florence Health Literacy Research Group. Measuring health literacy in Italy: The validation study of the HLS-EU-Q16 and of the HLS-EU- Q6 in Italian language, conducted in Florence and its surroundings. Ann. Ist. Super. Sanita.

[54] Rozin P. (2002). Human food intake and choice from biological, psychological, and cultural perspectives. Food Selection: From Genes to Culture.

[55] Freedman D.A., Bess K.D., Tucker H.A., Boyd D.L., Tuchman A.M., Wallston K.A. (2009). Public health literacy defined. Am. J. Prev. Med..

[56] Bröder J., Okan O., Bauer U., Bruland D., Schlupp S., Bollweg T.M., Saboga-Nunes L., Bond E., Sørensen K., Bitzer E.-M. (2017). Health literacy in childhood and youth: A systematic review of definitions and models. BMC Public Health.

