# Supplementary material for: Insights into the Predictors of Attitude toward Entomophagy: The Potential Role of Health Literacy: A Cross-Sectional Study Conducted in a Sample of Students of the University of Florence
Source: Int J Environ Res Public Health. 2021 May 17;18(10):5306. doi: 10.3390/ijerph18105306 (PMC8156530; doi:10.3390/ijerph18105306)
Supplement: Supplementary file 1 [file ijerph-18-05306-s001.zip › ijerph-1159378-supplementary.pdf]

Questionario sulle abitudini alimentari

Il seguente questionario è anonimo e i dati saranno analizzati solo in forma aggregata ed utilizzati solo per analisi statistiche ai fini di ricerca scientifica. Le chiediamo gentilmente pochi minuti per compilare questa scheda.

CORSO DI LAUREA: ____

SESSO: □ Maschio □ Femmina **ETÀ**:________________

PAESE DI ORIGINE: □ Italia □ Altro Se altro, quale? ______________________

ABITUDINI ALIMENTARI: □ Onnivoro □ Vegetariano □ Vegano

A. Hai mai sentito parlare di entomofagia?

□ Sì □ No

B. Dove hai sentito parlare di entomofagia?

□ Eventi gastronomici
□ Università
□ Mass-media
□ Altro

Se altro, quali? ____

C. Sai che esiste una tradizione culinaria di entomofagia in alcuni paesi del mondo?

□ Sì □ No

D. Quali vantaggi potrebbero derivare dal consumo di insetti?

COMPOSIZIONE NUTRIZIONALE: Per niente □ □ □ □ □ Tantissimo
IMPATTO AMBIENTALE: Per niente □ □ □ □ □ Tantissimo
ALTERNATIVA ALLA CARNE: Per niente □ □ □ □ □ Tantissimo
SAPORE GRADEVOLE: Per niente □ □ □ □ □ Tantissimo
FACILE REPERIBILITÀ: Per niente □ □ □ □ □ Tantissimo

E. Quali svantaggi potrebbero derivare dal consumo di insetti?

AVVERSITÀ CULTURALE: Per niente □ □ □ □ □ Tantissimo
REAZIONI ALLERGICHE: Per niente □ □ □ □ □ Tantissimo
PERICOLI MICROBIOLOGICI: Per niente □ □ □ □ □ Tantissimo
PERICOLI CHIMICI: Per niente □ □ □ □ □ Tantissimo

F. Hai mai mangiato insetti o prodotti a base di insetti?

□ Sì □ No Se Sì, quali? ____

G. Perché potresti iniziare a mangiare di insetti?

□ Necessità
□ Tradizioni locali / abitudini
□ Per provare gusti diversi
□ Caratteristiche nutrizionali
□ Altre ragioni
□ Non ci proverei mai

Se per altre ragioni, quali? ____

H. Da 0 a 4, quanto trovi disgustoso mangiare insetti?

Per niente □ □ □ □ □ Tantissimo

I. Come preferisci mangiare gli insetti?
□ Insetti Interi
□ Insetti sotto forma di farina
□ In entrambi i modi
□ In nessun modo

L. Consiglieresti ad altri di provare insetti o prodotti a base di insetti?

□ Sì □ No

M. Secondo te, quale organo di vigilanza dovrebbe essere responsabile del controllo della produzione di insetti per il consumo umano?

□ Stessa autorità assegnata come supervisore per i prodotti alimentari per il consumo umano
□ Ai produttori/associazioni di produttori
□ Ufficiali internazionali
□ Non lo so

N- HLS-EU-Q6

1. Secondo lei, quanto è difficile capire le informazioni riferite dai mezzi di comunicazione su come migliorare il suo stato di salute?

□ Molto difficile
□ Abbastanza difficile
□ Abbastanza facile
□ Molto facile
□ Non so

2. Secondo lei, quanto è difficile giudicare se le informazioni, sui rischi per la salute, riferite dai mezzi di comunicazione siano attendibili?

□ Molto difficile
□ Abbastanza difficile
□ Abbastanza facile
□ Molto facile

□ Non so

3. Secondo lei, quanto è difficile giudicare quali comportamenti quotidiani hanno un impatto sulla sua salute?

□ Molto difficile
□ Abbastanza difficile
□ Abbastanza facile
□ Molto facile
□ Non so

4. Secondo lei, quanto è difficile capire i consigli da parte di familiari o amici riguardo alla salute?

□ Molto difficile
□ Abbastanza difficile
□ Abbastanza facile
□ Molto facile
□ Non so

5- Secondo lei, quanto è difficile usare le informazioni che il medico le fornisce per prendere delle decisioni a proposito di una sua malattia?

□ Molto difficile
□ Abbastanza difficile
□ Abbastanza facile
□ Molto facile
□ Non so

6- Secondo lei, quanto è difficile capire quando è necessaria una seconda opinione da parte di un altro medico?

□ Molto difficile
□ Abbastanza difficile
□ Abbastanza facile
□ Molto facile
□ Non so
